# Social determinants of diabetes-related foot disease among older adults in New South Wales, Australia: evidence from a population-based study

**DOI:** 10.1186/s13047-021-00501-8

**Published:** 2021-12-16

**Authors:** Moin Uddin Ahmed, Wadad Kathy Tannous, Kingsley Emwinyore Agho, Frances Henshaw, Deborah Turner, David Simmons

**Affiliations:** 1 Translational Health Research Institute, School of Medicine, Western Sydney University, Campbelltown Campus, Locked Bag 1797, Penrith, NSW 2571 Australia; 2 Department of Economics, Finance and Property, School of Business, Western Sydney University, Parramatta Campus, Locked Bag 1797, Penrith, NSW 2571 Australia; 3 School of Health Sciences, Western Sydney University, Campbelltown Campus, Locked Bag 1797, Penrith, NSW 2571 Australia; 4 African Vision Research Institute (AVRI), University of KwaZulu-Natal, Durban, 4041 South Africa; 5 ConvaTec, Building 5, Brandon Business Park, 530 Springvale Rd, Glen Waverley, VIC 3150 Australia; 6 School of Clinical Sciences, Podiatric Medicine, Kelvin Grove Campus, Queensland University of Technology, Brisbane, QLD 4059 Australia; 7 Macarthur Clinical School, Western Sydney University, Campbelltown, NSW 2560 Australia

**Keywords:** Diabetic foot, Social determinants of health, Older adults, Australia, 45 and Up Study, Linked data

## Abstract

**Background:**

Diabetes-related foot is the largest burden to the health sector compared to other diabetes-related complications in Australia, including New South Wales (NSW). Understanding of social determinants of diabetes-related foot disease has not been definitive in Australian studies. This study aimed to investigate the social determinants of diabetes-related foot disease in NSW.

**Methodology:**

The first wave of the 45 and Up Study survey data was linked with NSW Admitted Patient Data Collection, Emergency Department Data Collection, and Pharmaceutical Benefits Scheme data resulting in 28,210 individuals with diabetes aged 45 years and older in NSW, Australia. Three outcome variables were used: diabetes-related foot disease (DFD), diabetic foot ulcer (DFU), and diabetic foot infection (DFI). They were classified as binary, and survey logistic regression was used to determine the association between each outcome measure and associated factors after adjusting for sampling weights.

**Results:**

The prevalence of DFD, DFU and DFI were 10.8%, 5.4% and 5.2%, respectively, among people with diabetes. Multivariate analyses revealed that the common factors associated with DFD, DFU and DFI were older age (75 years or more), male, single status, background in English speaking countries, and coming from lower-income households (less than AUD 20,000 per year). Furthermore, common lifestyle and health factors associated with DFD, DFU, and DFI were low physical activity (< 150 min of moderate-to-vigorous physical activity per week), history of diabetes for over 15 years, and having cardiovascular disease.

**Conclusion:**

Our study showed that about 1 in 10 adults with diabetes aged 45 years and older in NSW reported DFD. Interventions, including the provision of related health services aimed at reducing all forms of DFD in NSW, are recommended to target older individuals with a long history of diabetes, and coming from lower-income households.

**Supplementary Information:**

The online version contains supplementary material available at 10.1186/s13047-021-00501-8.

## Background

Diabetes-related foot disease (DFD) is defined as the presence of infection, ulceration or destruction of tissues of the foot associated with neuropathy and/or peripheral artery disease in the lower extremity of a person with diabetes mellitus [1]. DFD is a major public health concern due to substantial healthcare resources use, costs to individuals, the health system and society, and its negative effect on the quality of life [2]. Globally, the estimated number of people with DFD is 20 million [3], with an estimated 2 million people requiring lower limb amputation procedures [4]. Systematic reviews and meta-analyses suggest that the global prevalence of DFD is about 4.6–4.8% of the total population with diabetes [5, 6]. For Australia, the prevalence has been reported to be 1.5%, with DFD being responsible for 5400 lower limb amputations annually [6, 7].

DFD is one of the leading causes of hospitalisation globally [3, 8], with individuals with DFD having a higher risk in comparison with congestive heart failure, cerebrovascular disease, chronic renal failure, and chronic obstructive pulmonary disease [9]. In Australia, people with DFD have the highest number of hospital bed days among all diabetic complications [10], with an average length of stay in the hospital of 26 days due to lower limb amputation [11]. This compares to 8.2 days for diabetes patients with metabolic or cardiovascular disease, 3.4 days for heart failure and shock patients, and 2.7 days for patients with chronic obstructive airways disease [12, 13]. Diabetes-related foot ulcers and lower-limb wounds are not only the cause of lower-limb amputations but also deaths. In 2005, the most recent available death data for this condition, over 1000 deaths were due to foot ulcer and lower limb wounds which were about 8% of all diabetes attributed deaths [11]. Armstrong et al. (2013) concluded that people with diabetes-related foot ulcers (DFUs) have similar rates of morbidity and mortality as individuals with aggressive forms of cancer [14].

The clinical factors associated with diabetes-related foot complications are well researched, but there is a paucity of research for non-clinical factors. Non-clinical factors include social determinants of health (SDH). Research suggests that socioeconomic factors such as income, wealth, and education play an important role in a wide range of health outcomes [15]. According to the World Health Organization (WHO), the socioeconomic circumstances in which individuals are born, live, and work is the single most significant predictor of good or poor health [16]. Among different SDH, some of the key determinants recognised by the Australian Institute of Health and Welfare are socioeconomic status and educational attainment [17]. These determinants along with behavioural and psychological factors are not extensively researched for DFD in the Australian setting.

There are a limited number of Australian studies that investigated the association of DFD and social determinants. Bergin et al. (2011) suggested that the study used hospital separation data from Victoria to assess the relationship between diabetic foot morbidity and socioeconomic status [18], and Singh (2018) investigated the association of DFD and socioeconomic, geographic, and indigenous status using a representative inpatient population data from 2005 to 2011 in Queensland [19]. Again, in another study based on Queensland inpatient population in 2013, Lazzarini et al. (2017) investigated the social risk factors of peripheral arterial disease, peripheral neuropathy, and foot deformity for patients with and without diabetes [20]. The research reported by Perrin et al. (2019) included a wider range of social factors and performed a bivariate analysis of social factors and diabetic foot in a regional and rural area of Victoria and Tasmania [21]. Tapp et al. (2003) used a representative Australian population to analyse the association of social factors of DFD for persons with type-2 diabetes [22]. Existing studies suggest a scope of investigation of the association of social factors and DFD by including factors such as household income and private health insurance status. There is also a gap in the literature in terms of exploring the social factors of DFD in NSW- the largest state in Australia in terms of population and geographical area with the highest proportion (35%) of people with diabetes as a proportion of the total Australian population with diabetes [23].

This study sought to address the knowledge gap in understanding of social factors of diabetic foot complications by exploring the social determinants of DFD using a representative population of NSW during 2006–2012. The primary objective of this study is to investigate the likelihood of experiencing DFD, diabetic foot ulcer (DFU) and diabetic foot infection (DFI) based on social factors such as educational attainment, household income, private health insurance status and Index of Relative Socioeconomic Disadvantage. The secondary objective includes exploring associations of diabetic foot with demographic, lifestyle, and health status factors.

### Ethical clearance

This study has three ethics approvals: NSW Population and Health Services Research Ethics Committee (HREC/13/CIPHS/8), Western Sydney University Human Research Ethics Committee (H12215), and ACT Health Human Research Ethics and Governance (ETHLR.12.173). The 45 and Up Study was approved by the University of New South Wales Human Research Ethics Committee.

## Methods

### Data source

This study used data from participants of the 45 and Up Study baseline survey that was conducted between 2006 and 2009 in NSW. The 45 and Up Study was managed by the Sax Institute. About 267,153 respondents were randomly chosen from the Medicare Australia database, national health care records of citizens and permanent residents along with some temporary residents and refugees, who were aged 45 years and older, and had consented to have their medical data linked. This has been detailed elsewhere [24]. The 45 and Up Study is considered the largest population-based cohort study in Australia and the Southern hemisphere. The 45 and Up Study survey responses include self-reported data on participants’ demographic, socioeconomic, lifestyle and health factors.

For our study, the 45 and Up Study survey data were linked with health administrative databases (detailed below) by the Centre for Health Record Linkage (CHeReL) and the Sax Institute, two external agencies that provided the data for each participant deidentified through the use of a person project number (PPN) [25]. The data was accessed through the Secure Unified Research Environment (SURE), a cloud-based platform with a two-step authentication process.

The health administrative datasets linked to the survey data were NSW Admitted Patient Data Collection (APDC), NSW Emergency Department Data Collection (EDDC), and Pharmaceutical Benefits Scheme (PBS). NSW APDC data consists of hospital utilisation data on admitted patients to public hospitals, public psychiatric hospitals, public multi-purpose services, private hospitals, and private day procedures centres between 1999 to 2012 in NSW. EDDC consists of health service data on individuals presented at emergency departments of public hospitals in NSW between 2005 to 2012. In our study, APDC and EDDC of 2006–2012 were used to identify DFD, DFU and DFI. The PBS contains individuals’ data on prescribed pharmaceutical medications from 2004 to 2011. The PBS data were used to classify types of diabetes among survey participants. The CHeReL linked the 45 and Up Study data with APDC and EDDC using a probabilistic matching procedure, whereas the Sax Institute facilitated the linkage of the 45 and Up Study and PBS data using a deterministic matching procedure.

### Sample size and time-period

**Fig. 1:**
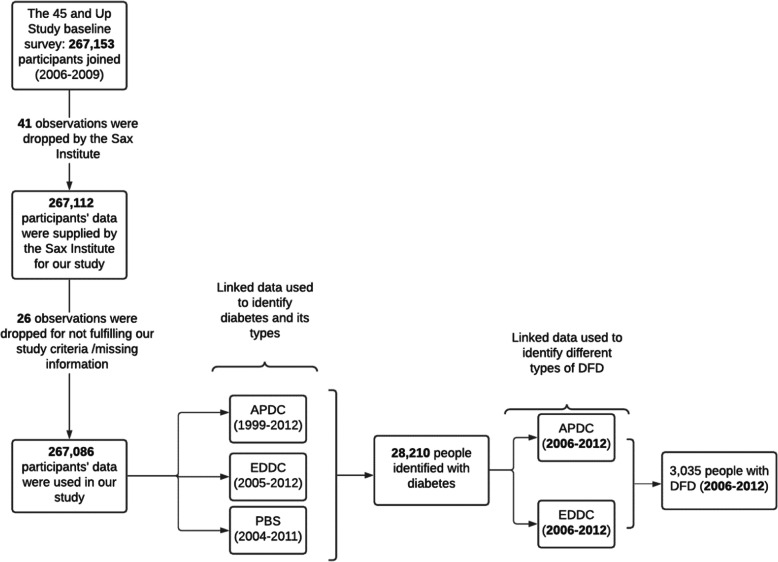
Participants of the current study, time period and data sources

The analytic sample of our study with time period is shown in Fig. 1. Out of 267,153 survey participants, 41 observations were dropped by the Sax Institute due to ongoing maintenance and data cleaning. Our study was supplied with the data of 267,112 participants. A further 26 observations were dropped whose age was less than 45 years old or had missing information. Among 267,086 participants, our study identified 28,210 people with diabetes, and it was the size of our analytic sample. Using this sample, our study utilised APDC and EDDC from 2006 to 2012 to identify people DFD, DFU and DFI.

### Identification of diabetes, DFD, DFU and DFI

**Fig. 2:**
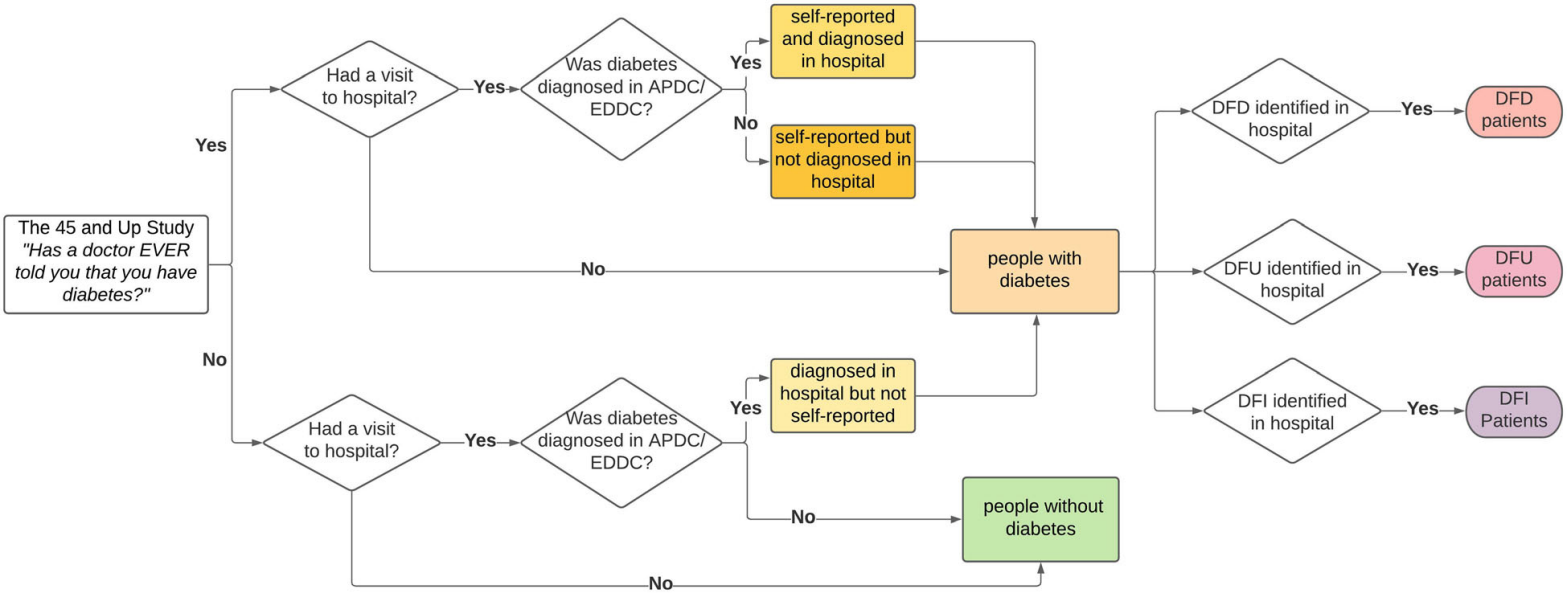
Identification of people with diabetes, DFD, DFU and DFI from the 45 and Study survey data and linked health administrative data

The term “diabetes-related foot complication” or “diabetic foot complication” was used to refer to any DFD, DFU and DFI conditions in this paper. The identification process of people with diabetes and diabetic foot complications is detailed in Fig. 2. In the first step, diabetes status was determined based on either self-reported data or hospital diagnostic codes [22]. The participants of the 45 and Up Study survey participants were asked, “Has a doctor ever told you that you have diabetes?”. If the answer was “yes”, then they were assigned to have diabetes. In addition, participants belonged to the diabetes group if they were diagnosed with diabetes in the hospital during their hospital admissions or emergency department presentations. Diagnosis codes used in this study were International Classification of Diseases 9 - Clinical Modification (ICD-9 CM), International Classification of Diseases 10 - Australian Modification (ICD-10 AM) and Systematized Nomenclature of Medicine - Clinical Terms (SNOMED- CT). This was due to the different sources of data and the timeframe. The complete list of related diagnostic codes used for this study is presented in Supplementary Table 1 and Supplementary Table 2 [18, 26–30].

In the second step, the identification of diabetes-related foot complications was ascertained only if the foot complication was identified at the same time or after diabetes identification (Fig. 2).

### Population and outcome definition

The analytic sample consists of 28,210 individuals whose diabetic foot complications status were observed in APDC and EDDC from 2006 to 2012. The study outcome variables were DFD, DFU and DFI, which were dichotomised. DFD included the ulcer of foot or lower limb, decubitus ulcer, peripheral angiopathy with or without gangrene, cellulitis of toe or lower limb, osteomyelitis, mono/polyneuropathy of lower limb, neuropathic arthropathy, and diabetes-related amputation of the lower limb [1, 18, 19, 31]. DFU included ulcer of foot or lower limb, decubitus ulcer, whereas DFI included cellulitis or osteomyelitis of foot or lower limb [1, 32, 33].

### Study variables

Our choice of study variables was based on similar studies that examined the relationship between DFD (or diabetes) and other factors [19–22, 34–40]. For instance, the study by Lazzarini et al. (2017) included age, sex, socioeconomic status, education level, smoking status and depression while investigating the associated factors of foot complications in a representative inpatient population of Australia [20]. They found that age and socioeconomic status were significantly associated with a previous foot ulcer. Tapp et al. (2002) concluded that diabetes duration and hypertension were predictors of peripheral vascular disease in an Australian population-based study [22]. The study by Nather et al. (2010) demonstrated that household income and physical activity to be significantly associated with diabetic foot in an inpatient study in Singapore [37]. Study variables of our study were classified into demographic, socioeconomic, lifestyle risk factors, and health status variables.

Demographic variables included sex, age, marital status, remoteness of residence, country of birth, and whether they spoke a language other than English. Socioeconomic variables included the highest education level, socioeconomic status, household income, and private health insurance status. These variables were categorised following previous Australian studies [20, 37, 41–43]. To mention a few, age was categorised into four groups: 45–54 years, 55–64 years, 65–74 years, and 75 years or older following the 45 and Up Study related diabetes studies [41, 42, 44]. The highest attained educational qualification had four groups: less than high school, high school certificate/trade, certificate/diploma, and university and higher [43]. Socioeconomic status was assessed by the Australian Bureau of Statistics’ index of relative socio-economic disadvantage (IRSD) [45]. In our study, IRSD scores (the highest proportion of disadvantage) was ranked in quantiles, with lower scores indicating higher levels of social disadvantage [43]. Annual household income (in AUD) was classified into three categories: less than AUD 20,000, AUD 20,000 to less than AUD 50,000, and AUD 50,000 and over [41, 44]. The private health insurance variable was categorised into three groups: “No (without Department of Veterans’ Affairs (DVA) card and concession card)”, “No (with DVA card or concession card) and “Yes”. The current or former members of the Australian Defence Force or their dependents are eligible for DVA card, whereas eligible people can access the concession card. The eligibility of concession cards is determined by many factors, including age, income, and disability status. DVA or concession cardholders can receive more subsidised health services and medicine compared to non-holders [46, 47].

Lifestyle risk factor variables included self-reported smoking status, alcohol consumption, physical activity, and fruits and vegetables intake. The classification followed previous studies or guidelines [48–50]. Health status variables included the duration of diabetes, body mass index (BMI), presence of different comorbidities (high blood pressure, high blood cholesterol, heart disease, stroke, asthma, and psychological distress). BMI was calculated from self-reported height and weight and was categorised according to the National Health and Medical Research Council guidelines [51]. Psychological distress, measured using the Kessler-10 (K10) instrument [52] were classified into two groups: “None/low/moderate” and “High/very high” [43].

### Statistical analysis

Data analysis was performed using the ‘*svy*’ command in Stata version 16 to allow for adjustments of sampling weights. The preliminary analysis included the frequency tabulation of all study factors included in the study. A Venn diagram was also produced for all the outcome variables.

Survey logistic regression adjusted for survey weights to determine the association between each outcome measure and different risk factors. In the first step, univariate analysis was performed to examine the unadjusted odds ratio (OR). Next, multivariate logistic regression models were employed to examine the association between each outcome variable and study factors. Adjusted Odds ratios (AORs) with 95% confidence interval (CI) were calculated to measure the association of the study variables and DFD and its different types.

The potential impact of missing data was considered on the estimated coefficients in sensitivity analyses with the imputed dataset. The chain equation method was applied for the multiple imputations, assuming that data were missing at random [53]. The approach also assumed that available information on the participants’ characteristics could be used to investigate the participants with missing data [54]. All study factors and outcome variables of the main analysis were considered in the multiple imputation models. The imputation was conducted using Stata 16 with ‘*mi*’ command. The sensitivity analysis was conducted based on 25 imputations [55], and revised AORs with 95% CI were presented to compare with complete case analysis.

## Results

### Characteristics of the study population

The present study included 28,210 people with diabetes aged 45 years and older in NSW, Australia from the 45 and Up Study data. The number of people identified with DFD was 3035 during 2006–2012. Among 3035 individuals with DFD, 838 individuals were diagnosed with DFU only, 783 individuals were diagnosed with DFI only, 665 persons had both DFU and DFI, while 741 persons had other types of DFD (peripheral vascular disease, peripheral neuropathy, mononeuropathy, and diabetic neuropathic arthropathy).

**Fig. 3:**
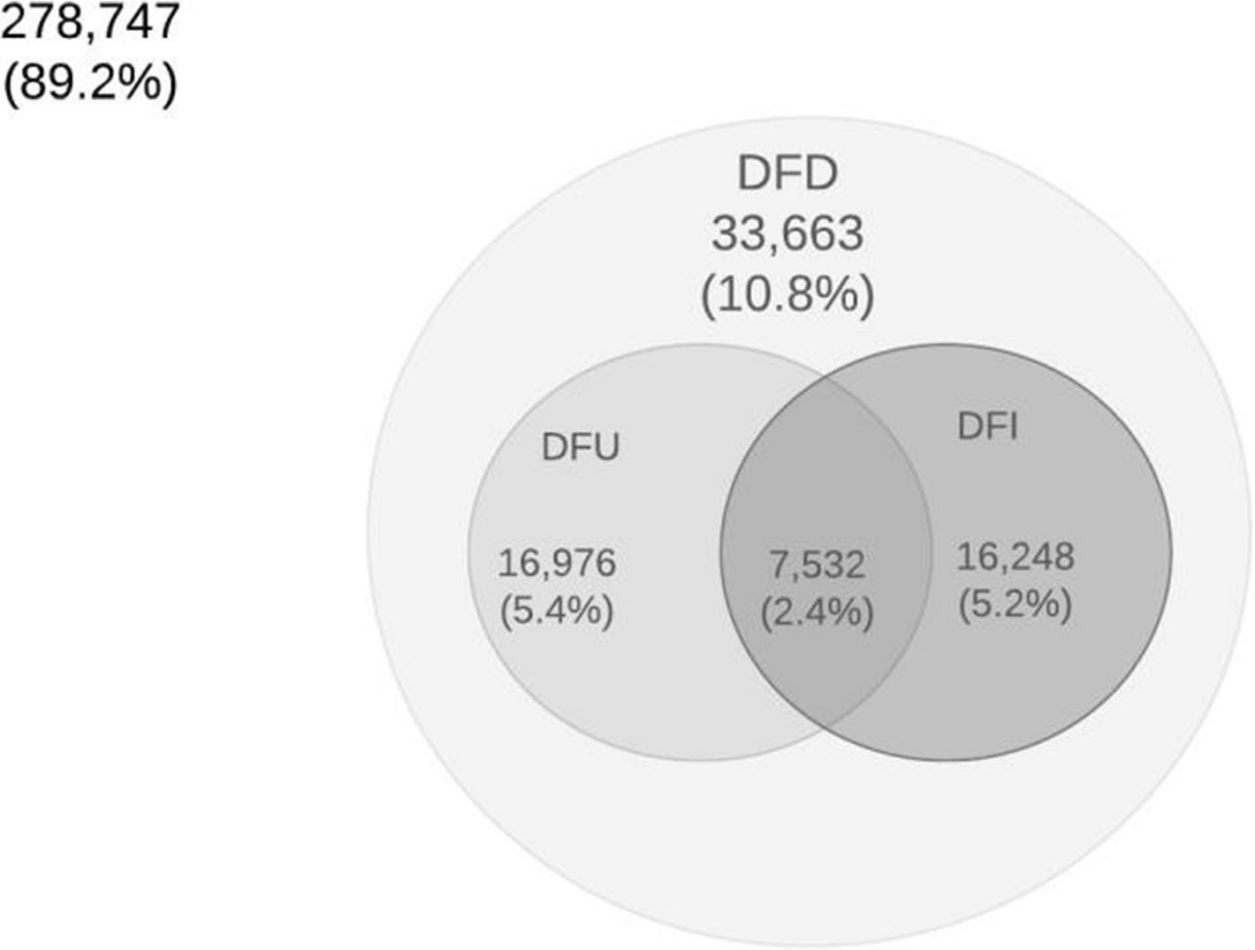
Venn Diagram of DFD (*N* = 33,663) in terms of DFU (*N* = 16,976) and DFI (*N* = 16,248) for people with diabetes (*N* = 312,410) in NSW during 2006–2012; N: Total weighted frequency

The number of people with DFD, DFU and DFI was also estimated at the population level (weighted frequency). The total number of DFD patients were estimated as 33,663 in NSW during 2006–2012 at the population level, whereas the estimated number of DFU and DFI patients were 16,976 and 16,248, respectively (Fig. 3). As shown in Fig. 3, there was an overlap between DFU and DFI (2.4%) adults with diabetes aged 45 years and older who lived in NSW. Again, 10.8% of adults with diabetes aged 45 years and older from NSW had DFD, 5.4% had DFU, and 5.2% had DFI (Fig. 3).

Participants’ demographic, socioeconomic, health status, and lifestyle characteristics (weighted frequency and weighted percentage) is presented in Table 1. A large number of participants (40–45%) with DFD, DFU and DFI belonged to the “75 years or older age group” while most were from English-speaking countries (75–80%). About two-thirds of the participants were from households with an annual income of less than AUD 20,000 for all types of foot complications. Regarding lifestyle factors, about 55% of survey participants were reported to be ever being a regular smoker, and 54–58% of participants performed less than 150 min of moderate-to-vigorous physical activity per week. In regard to health status factors, it was found that 39–50% of diabetic foot patients had diabetes for 15 years or more, and around 65% of patients suffered cardiovascular diseases.

**Table 1:** Characteristics of the study participants with DFD, DFU and DFI

	Diabetes-related foot disease (***N*** = 33,663)		Diabetic foot ulcer (***N*** = 16,976)		Diabetic foot infection (***N*** = 16,248)	
	n	%	n	%	n	%
**Demographic factors**						
**Age**						
45–54 years	2989	8.9%	1309	7.7%	1718	10.6%
55–64 years	6428	19.1%	2963	17.5%	3645	22.4%
65–74 years	9017	26.8%	4025	23.5%	4077	25.1%
75+ years	15,229	45.2%	8678	51.1%	6808	41.9%
**Sex**						
Male	19,365	57.5%	9665	56.9%	9166	56.4%
Female	14,298	42.5%	7311	43.1%	7082	43.6%
**Current marital status (*N*** **= 33,422, 16,862, 16,155)**						
Single	2188	6.5%	1281	7.6%	1425	8.8%
Married/defacto	16,582	49.6%	7800	46.3%	7530	46.6%
Widowed	8791	26.3%	4831	28.7%	4370	27.0%
Divorced/separated	5861	17.5%	2949	17.5%	2831	17.5%
**Remoteness**						
Major cities	20,399	60.6%	10,611	62.5%	9744	60.0%
Inner regional	8254	24.5%	4084	24.1%	3795	23.4%
Outer regional	4158	12.4%	1868	11.0%	2235	13.8%
Remote	817	2.4%	376	2.2%	448	2.8%
Very remote	36	0.1%	36	0.2%	26	0.2%
**Country of birth (*N*** **= 33,043, 16,680, 15,982)**						
English speaking countries	24,749	74.9%	12,735	76.3%	12,207	76.4%
Europe	6742	20.4%	3329	20.0%	3026	18.9%
Middle East	296	0.9%	145	0.9%	151	0.9%
Asia	627	1.9%	248	1.5%	344	2.2%
Others	629	1.9%	223	1.3%	253	1.6%
**Language spoken other than English**						
No	28,930	85.9%	14,711	86.7%	13,974	86.0%
Yes	4733	14.1%	2265	13.3%	2275	14.0%
**Socio-economic factors**						
**Education (*N*** **= 32,264, 16,129, 15,571)**						
Less than high school	16,838	52.2%	8580	53.2%	8501	54.6%
High school certificate/ trade	7534	23.4%	4083	25.3%	3089	19.8%
Certificate/diploma	4474	13.9%	2026	12.6%	2314	14.9%
University degree or higher	3418	10.6%	1440	8.9%	1667	10.7%
**SEIFA (IRSD)**						
quantile 1 (least disadvantaged)	9132	27.1%	4615	27.2%	4578	28.2%
quantile 2	6323	18.8%	3285	19.4%	3264	20.1%
quantile 3	6686	19.9%	3372	19.9%	3010	18.5%
quantile 4	6704	19.9%	3257	19.2%	3005	18.5%
quantile 5 (most disadvantaged)	4819	14.3%	2448	14.4%	2391	14.7%
**Annual household income (*N*** **= 23,711, 11,817, 11,506)**						
< AUD 20,000	14,064	59.3%	7154	60.5%	6907	60.0%
AUD 20,000 - < AUD 50,000	6471	27.3%	3215	24.2%	2898	25.2%
AUD 50,000 or more	3176	13.4%	1448	12.3%	1701	14.8%
**Private health insurance**						
No (without DVA/concession card)	5342	15.9%	2549	15.0%	2882	16.8%
No (with DVA/concession card)	15,482	46.0%	8185	48.2%	7554	46.5%
Yes	12,839	38.1%	6243	36.8%	5812	35.8%
**Life-style factors**						
**Ever being a regular smoker (*N*** **= 33,632, 16,950, 16,217)**						
No	14,821	44.1%	7622	45.0%	7595	46.8%
Yes	18,811	55.9%	9329	55.0%	8623	53.2%
**Alcohol consumption (*N*** **= 32,102, 16,142, 15,505)**						
< = 14 drinks per week	29,047	90.5%	14,658	90.8%	14,043	90.6%
> 14 drinks per week	3055	9.5%	1485	9.2%	1462	9.4%
**Total moderate-to-vigorous physical activity per week (minutes)**						
< 150 min	18,194	54.0%	9889	58.3%	8790	54.1%
150–300 min	4076	12.1%	1997	11.8%	1894	11.7%
> 300 min	11,393	33.8%	5090	30.0%	5564	34.2%
**Vegetables intake (*N*** **= 32,065, 16,077, 15,384)**						
< 5 serves per day	21,699	67.7%	10,916	67.9%	10,188	66.2%
5 or more serves per day	10,365	32.3%	5161	32.1%	5196	33.8%
**Fruits intake (*N*** **= 33,126, 16,711, 15,912)**						
< 2 serves per day	13,923	42.0%	7191	43.0%	6623	41.6%
2 or more serves per day	19,204	58.0%	9520	57.0%	9289	58.4%
**Health factors**						
**Type of diabetes**						
Type-1	1690	5.0%	845	5.0%	884	5.4%
Type-2	31,973	95.0%	16,131	95.0%	15,364	94.6%
**Duration of diabetes in years (*N*** **= 23,206, 11,370, 11,145)**						
< 5 years	4671	20.1%	2037	17.9%	2615	23.5%
5 to < 10 years	5228	22.5%	2272	20.0%	2449	22.0%
10 to < 15 years	4374	18.8%	2000	17.6%	1907	17.1%
15 years or more	8933	38.5%	5061	44.5%	4174	37.5%
**BMI classification (*N*** **= 33,002, 16,587, 15,893)**						
< 18.5	509	1.5%	334	2.0%	207	1.3%
18.5 to less than 25	6810	20.6%	4026	24.3%	2833	17.8%
25 to less than 30	9810	29.7%	4316	26.0%	4246	26.7%
30 or more	15,872	48.1%	7911	47.7%	8607	54.2%
**High blood pressure**						
No	15,462	45.9%	8210	48.4%	7640	47.0%
Yes	18,201	54.1%	8766	51.6%	8609	53.0%
**High blood cholesterol**						
No	24,666	73.3%	12,695	74.8%	12,015	73.9%
Yes	8997	26.7%	4281	25.2%	4234	26.1%
**Cardiovascular disease**						
No	11,294	33.5%	5806	34.2%	5813	35.8%
Yes	22,369	66.5%	11,170	65.8%	10,435	64.2%
**Stroke**						
No	29,349	87.2%	14,803	87.2%	14,207	87.4%
Yes	4314	12.8%	2173	12.8%	2041	12.6%
**Asthma**						
No	28,216	83.8%	14,518	85.5%	13,091	80.6%
Yes	5447	16.2%	2458	14.5%	3158	19.4%
**Psychological distress**						
None/low/moderate	28,857	85.7%	14,777	87.0%	13,891	85.5%
High/very high	4806	14.3%	2199	13.0%	2358	14.5%

n is weighted frequency; N is total weighted frequency (mentioned in brackets for variables with missing values); % denotes weighted percentage

*SEIFA* Socio-Economic Indexes for Areas, *IRSD* The Index of Relative Socio-Economic Disadvantage, *DVA* Department of Veterans’ Affairs, Psychological distress, None/low/moderate: less than 22 Kessler-10 (K10) score

### Determinants of DFD

The association of study factors and DFD in terms of unadjusted and adjusted ORs from logistic regression with 95% CI was presented in Table 2. Regarding demographic factors, the study showed that older people (65–74 years and 75 years or over), males, single individuals, people from English-speaking countries were significantly more likely to have DFD. Individuals from remote areas had an 82% higher likelihood of experiencing DFD than those in major cities (*p* = 0.007). Individuals with an annual household income of AUD 20,000 to less than AUD 50,000 had significantly lower likelihood (AOR = 0.83, 95% CI: 0.70, 0.98) of having DFD compared to individuals with less than AUD 20,000 household income. The odds of having DFD was also found to be lower for people with income of AUD 50,000 or more (AOR = 0.59, 95% CI: 0.46, 0.76).

**Table 2:** Association between study factors and DFD: unadjusted and adjusted odds ratios

Study factors	OR	95% CI	***p*** value	AOR	95% CI	***p*** value
**Demographic factors**						
**Age**						
45–54 years	1.00			1.00		
55–64 years	1.40	(1.10–1.79)	0.007	1.24	(0.94–1.65)	0.129
65–74 years	2.01	(1.59–2.54)	< 0.001	1.63	(1.22–2.18)	0.001
75+ years	3.95	(3.14–4.96)	< 0.001	2.85	(2.10–3.85)	< 0.001
**Sex**						
Male	1.00			1.00		
Female	0.82	(0.73–0.93)	0.002	0.69	(0.58–0.81)	< 0.001
**Current marital status**						
Single	1.00			1.00		
Married/defacto	0.60	(0.48–0.74)	< 0.001	0.63	(0.48–0.82)	0.001
Widowed	1.12	(0.87–1.43)	0.371	0.69	(0.51–0.94)	0.020
Divorced/separated	0.84	(0.64–1.09)	0.185	0.83	(0.60–1.14)	0.256
**Remoteness**						
Major cities	1.00			1.00		
Inner regional	0.99	(0.87–1.12)	0.846	0.92	(0.79–1.09)	0.344
Outer regional	1.12	(0.96–1.31)	0.139	1.03	(0.83–1.27)	0.793
Remote	1.78	(1.27–2.50)	0.001	1.82	(1.18–2.81)	0.007
Very remote	1.01	(0.33–3.05)	0.986	1.05	(0.28–3.99)	0.939
**Country of birth**						
English speaking countries	1.00			1.00		
Europe	0.99	(0.86–1.15)	0.944	0.81	(0.66–1.00)	0.047
Middle East	0.26	(0.12–0.60)	0.002	0.21	(0.08–0.51)	0.001
Asia	0.26	(0.16–0.42)	< 0.001	0.31	(0.16–0.58)	< 0.001
Others	0.48	(0.30–0.77)	0.002	0.49	(0.28–0.86)	0.013
**Socio-economic factors**						
**SEIFA (IRSD)**						
quantile 1 (least disadvantaged)	1.00			1.00		
quantile 2	1.08	(0.90–1.29)	0.410	0.95	(0.77–1.18)	0.651
quantile 3	1.01	(0.85–1.21)	0.894	1.06	(0.85–1.32)	0.621
quantile 4	1.04	(0.86–1.24)	0.707	1.30	(1.04–1.63)	0.021
quantile 5 (most disadvantaged)	1.08	(0.90–1.30)	0.389	1.48	(1.16–1.89)	0.002
**Annual household income**						
< AUD 20,000	1.00			1.00		
AUD 20,000 - < AUD 50,000	0.66	(0.58–0.75)	< 0.001	0.83	(0.70–0.98)	0.027
AUD 50,000 or more	0.36	(0.30–0.42)	< 0.001	0.59	(0.46–0.76)	< 0.001
**Life-style factors**						
**Ever being a regular smoker**						
No	1.00			1.00		
Yes	1.37	(1.22–1.55)	< 0.001	1.31	(1.13–1.51)	< 0.001
**Total moderate-to-vigorous physical activity per week (minutes)**						
< 150 min	1.00			1.00		
150–300 min	0.53	(0.44–0.63)	< 0.001	0.63	(0.51–0.78)	< 0.001
> 300 min	0.51	(0.45–0.57)	< 0.001	0.59	(0.50–0.68)	< 0.001
**Health status factors**						
**Type of diabetes**						
Type-1	1.00			1.00		
Type-2	0.65	(0.47–0.92)	0.014	0.62	(0.43–0.88)	0.008
**Duration of diabetes**						
< 5 years	1.00			1.00		
5 to < 10 years	1.48	(1.21–1.81)	< 0.001	1.36	(1.10–1.67)	0.004
10 to < 15 years	1.67	(1.36–2.05)	< 0.001	1.41	(1.15–1.74)	0.001
15 years or more	2.85	(2.36–3.43)	< 0.001	2.01	(1.66–2.45)	< 0.001
**BMI classification**						
< 18.5	1.00			1.00		
18.5 to less than 25	0.51	(0.30–0.89)	0.017	0.40	(0.20–0.79)	0.008
25 to less than 30	0.46	(0.27–0.78)	0.005	0.41	(0.21–0.79)	0.008
30 or more	0.54	(0.31–0.92)	0.024	0.51	(0.26–1.00)	0.050
**Cardiovascular disease**						
No	1.00			1.00		
Yes	2.09	(1.84–2.37)	< 0.001	1.62	(1.40–1.89)	< 0.001
**Stroke**						
No	1.00			1.00		
Yes	1.91	(1.60–2.30)	< 0.001	1.37	(1.09–1.73)	0.008
**Asthma**						
No	1.00			1.00		
Yes	1.24	(1.05–1.46)	0.012	1.26	(1.02–1.56)	0.034
**Psychological distress**						
None/low/moderate	1.00			1.00		
High/very high	1.27	(1.05–1.54)	0.013	1.30	(1.03–1.65)	0.029

*OR* Unadjusted Odds Ratio, *AOR* Adjusted Odds ratio, Coefficients, 95% CI and *p* values are reported for those variables which have at least one significant (*p* < 0.05) category, *SEIFA* Socio-Economic Indexes for Areas, *IRSD* The Index of Relative Socio-Economic Disadvantage, *DVA* Department of Veterans’ Affairs, Psychological distress, None/low/moderate: less than 22 Kessler-10 (K10) score

Smoking and physical activity were significantly associated with having DFD (*p* < 0.001). It was observed that the odds of experiencing DFD was higher among people who were ever being a regular smoker (AOR = 1.31, 95% CI: 1.13, 1.51) compared to those who were never a regular smoker (Table 2). Again, people who performed moderate-to-vigorous physical activity of 150 min or more per week had a lower likelihood of having DFD (AOR = 0.63, 95% CI: 0.51, 0.78 and AOR = 0.59, 95% CI: 0.50, 0.68). Health status factors associated with a higher likelihood of having DFD included type-1 diabetes, diabetes duration of 15 years or more, and lower BMI. Results also indicated that people were more likely to have DFD if they had cardiovascular disease (AOR = 1.62, 95% CI: 1.40, 1.89), had a stroke (AOR = 1.37, 95% CI: 1.09, 1.73), had high level of psychological distress (AOR = 1.30, 95% CI: 1.03, 1.65) as opposed to those who did not have these conditions.

### Determinants of DFU

The unadjusted and adjusted ORs for the association between DFU and study variables are shown in Table 3. With regards to demographic and socioeconomic factors, older age, male, single, individuals from English speaking countries, and patients from lower household income had a significantly higher likelihood of having DFU. Results showed that people aged 75 years and over (AOR = 4.19, 95% CI: 2.66, 6.58) were more likely to experience DFD than people from the 45–54 years age group. People who reported having an annual household income of more than AUD 50,000 (AOR = 0.58, 95% CI: 0.40, 0.84) had a lower likelihood of having DFU compared to people with a household income of less than AUD 20,000.

**Table 3:** Association between study factors and DFU: unadjusted and adjusted odds ratios

Study factors	OR	95% CI	***p*** value	AOR	95% CI	***p*** value
**Demographic factors**						
**Age**						
45–54 years	1.00			1.00		
55–64 years	1.89	(1.25–2.88)	0.003	1.62	(1.05–2.48)	0.027
65–74 years	2.57	(1.71–3.87)	< 0.001	1.84	(1.18–2.88)	0.007
75+ years	6.41	(4.34–9.46)	< 0.001	4.19	(2.66–6.58)	< 0.001
**Sex**						
Male	1.00			1.00		
Female	0.82	(0.68–1.01)	0.056	0.73	(0.58–0.92)	0.008
**Current Marital status**						
Single	1.00			1.00		
Married/defacto	0.53	(0.39–0.72)	< 0.001	0.55	(0.39–0.77)	0.001
Widowed	1.00	(0.70–1.43)	0.993	0.57	(0.39–0.85)	0.005
Divorced/separated	0.66	(0.45–0.98)	0.041	0.70	(0.47–1.06)	0.096
**Country of birth**						
English speaking countries	1.00			1.00		
Europe	0.97	(0.76–1.23)	0.779	0.76	(0.57–1.02)	0.064
Middle East	0.24	(0.07–0.80)	0.020	0.19	(0.05–0.70)	0.013
Asia	0.17	(0.08–0.37)	< 0.001	0.18	(0.08–0.44)	< 0.001
Others	0.28	(0.12–0.65)	0.003	0.25	(0.10–0.60)	0.002
**Socio-economic factors**						
**Annual household income**						
< AUD 20,000	1.00			1.00		
AUD 20,000 - < AUD 50,000	0.68	(0.56–0.84)	< 0.001	0.81	(0.64–1.04)	0.101
AUD 50,000 or more	0.31	(0.24–0.41)	< 0.001	0.58	(0.40–0.84)	0.004
**Life-style factors**						
**Ever being a regular smoker**						
No	1.00			1.00		
Yes	1.31	(1.09–1.58)	0.004	1.28	(1.05–1.56)	0.016
**Total moderate-to-vigorous physical activity per week (minutes)**						
< 150 min	1.00			1.00		
150–300 min	0.53	(0.41–0.70)	< 0.001	0.67	(0.50–0.89)	0.005
> 300 min	0.44	(0.36–0.54)	< 0.001	0.54	(0.44–0.67)	< 0.001
**Health factors**						
**Type of diabetes**						
Type-1	1.00			1.00		
Type-2	0.58	(0.35–0.95)	0.031	0.56	(0.33–0.94)	0.028
**Duration of diabetes**						
< 5 years	1.00			1.00		
5 to < 10 years	1.61	(1.20–2.16)	0.002	1.46	(1.08–1.97)	0.013
10 to < 15 years	1.98	(1.47–2.65)	< 0.001	1.62	(1.21–2.19)	0.001
15 years or more	3.85	(2.95–5.02)	< 0.001	2.54	(1.92–3.34)	< 0.001
**BMI classification**						
< 18.5	1.00			1.00		
18.5 to less than 25	0.37	(0.17–0.81)	0.013	0.39	(0.17–0.88)	0.024
25 to less than 30	0.26	(0.12–0.57)	0.001	0.29	(0.13–0.66)	0.003
30 or more	0.35	(0.16–0.77)	0.009	0.45	(0.20–1.02)	0.057
**Cardiovascular disease**						
No	1.00			1.00		
Yes	2.39	(1.96–2.92)	< 0.001	1.66	(1.34–2.05)	< 0.001

*OR* Unadjusted Odds Ratio, *AOR* Adjusted Odds ratio; Coefficients, 95% CI and *p* values are reported for those variables which have at least one significant (*p* < 0.05) category, *SEIFA* Socio-Economic Indexes for Areas, *IRSD* The Index of Relative Socio-Economic Disadvantage, *DVA* Department of Veterans’ Affairs, Psychological distress, None/low/moderate: less than 22 Kessler-10 (K10) score

Regarding lifestyle and health status factors, it was revealed that ever smoker, low level of physical activities, type-1 diabetes, longer duration of diabetes, lower BMI, and cardiovascular disease were significantly associated with DFU. It was found that an ever smoker was 28% more likely to experience DFU compared to people being never smokers (*p* = 0.016). People with type-2 diabetes were less likely to develop DFU (AOR = 0.56, 95% CI: 0.33, 0.948) than people with type-1 diabetes. The likelihood of having DFU was higher for people who reported having diabetes for 15 years or more (AOR = 2.54, 95% CI: 1.92, 3.34). The odds of having DFU was higher for people who reported experiencing cardiovascular disease (AOR = 1.66, 95% CI: 1.34, 2.05) than those who did not have the condition.

### Determinants of DFI

The unadjusted and adjusted ORs for the association between DFI and study factors were illustrated in Table 4. Demographic and socioeconomic factors associated with having DFI were older age, male, single status, coming from lower household income, and having no private health insurance. People aged 75 years or more (AOR = 1.93, 95% CI: 1.28, 2.90) were more likely to experience DFI than people of age group 45–54 years. The study found that people living in the most disadvantaged areas (AOR = 1.42, 95% CI: 1.02, 1.97) had a higher likelihood of suffering from DFI than people from the least disadvantaged group. It was also found that people with household income of AUD 50,000 (AOR = 0.62, 95% CI: 0.44, 0.87) had lower odds of having DFI than people with a household income of less than AUD 20,000. Again, people who had private health insurance (AOR = 0.71, 95% CI: 0.52, 0.94) were significantly less likely to have DFI compared to those who did not have private health insurance and DVA/concession card.

**Table 4:** Association between study factors and DFI: unadjusted and adjusted odds ratios

Study factors	OR	95% CI	***p*** value	AOR	95% CI	***p*** value
**Demographic factors**						
**Age**						
45–54 years	1.00			1.00		
55–64 years	1.30	(0.91–1.87)	0.154	1.17	(0.81–1.69)	0.393
65–74 years	1.56	(1.09–2.24)	0.015	1.26	(0.85–1.87)	0.254
75+ years	2.65	(1.87–3.76)	< 0.001	1.93	(1.28–2.90)	0.002
**Sex**						
Male	1.00			1.00		
Female	0.85	(0.69–1.04)	0.107	0.61	(0.49–0.77)	< 0.001
**Current Marital status**						
Single	1.00			1.00		
Married/defacto	0.41	(0.29–0.56)	< 0.001	0.47	(0.33–0.66)	< 0.001
Widowed	0.78	(0.54–1.13)	0.183	0.64	(0.43–0.94)	0.022
Divorced/separated	0.53	(0.35–0.80)	0.002	0.54	(0.36–0.82)	0.004
**Socio-economic factors**						
**SEIFA (IRSD)**						
quantile 1 (least disadvantaged)	1.00			1.00		
quantile 2	0.94	(0.71–1.24)	0.664	0.94	(0.70–1.25)	0.656
quantile 3	0.75	(0.56–1.01)	0.056	0.82	(0.60–1.12)	0.204
quantile 4	0.89	(0.67–1.20)	0.450	1.05	(0.77–1.43)	0.760
quantile 5 (Most disadvantaged)	1.04	(0.78–1.39)	0.793	1.42	(1.02–1.97)	0.035
**Annual household income**						
< AUD 20,000	1.00			1.00		
AUD 20,000 - < AUD 50,000	0.64	(0.52–0.80)	< 0.001	0.77	(0.60–0.98)	0.032
AUD 50,000 or more	0.40	(0.31–0.52)	< 0.001	0.62	(0.44–0.87)	0.005
**Private health insurance**						
No (without DVA/concession card)	1.00			1.00		
No (with DVA/concession card)	1.34	(1.01–1.78)	0.042	0.96	(0.71–1.28)	0.786
Yes	0.69	(0.57-0.84)	< 0.001	0.71	(0.52–0.94)	0.020
**Life-style factors**						
**Total moderate-to-vigorous physical activity per week (minutes)**						
< 150 min	1.00			1.00		
150–300 min	0.60	(0.45–0.79)	< 0.001	0.70	(0.53–0.93)	0.015
> 300 min	0.54	(0.44–0.66)	< 0.001	0.64	(0.52–0.80)	< 0.001
**Health status factors**						
**Type of diabetes**						
Type-1	1.00			1.00		
Type-2	0.57	(0.35–0.92)	0.020	0.55	(0.33–0.90)	0.018
**Duration of diabetes**						
< 5 years	1.00			1.00		
5 to < 10 years	1.23	(0.93–1.63)	0.149	1.13	(0.85–1.51)	0.386
10 to < 15 years	1.23	(0.91–1.65)	0.172	1.07	(0.80–1.44)	0.631
15 years or more	2.21	(1.71–2.86)	< 0.001	1.62	(1.25–2.10)	< 0.001
**Cardiovascular disease**						
No	1.00			1.00		
Yes	1.80	(1.47–2.20)	< 0.001	1.36	(1.11–1.67)	0.003
**Stroke**						
No	1.00			1.00		
Yes	2.03	(1.48–2.80)	< 0.001	1.40	(1.01–1.94)	0.043
**Asthma**						
No	1.00			1.00		
Yes	1.78	(1.38–2.30)	< 0.001	1.64	(1.26–2.15)	< 0.001

*OR* Unadjusted Odds Ratio, *AOR* Adjusted Odds ratio; Coefficients, 95% CI and *p* values are reported for those variables which have at least one significant (*p* < 0.05) category, *SEIFA* Socio-Economic Indexes for Areas, *IRSD* The Index of Relative Socio-Economic Disadvantage, *DVA* Department of Veterans’ Affairs, Psychological distress, None/low/moderate: less than 22 Kessler-10 (K10) score

Although the complete case analysis did not find any association of remoteness of residence area, country of birth and education with DFI (Table 4), the multiple imputation analysis suggested that the associations were significant (Supplementary Table 3). Results from multiple imputation analyses suggested that people residing in remote areas (AOR = 1.65, 95% CI: 1.05, 2.58) were more likely to have DFI than major city dwellers (Supplementary Table 3).

Among the lifestyle factors considered in this study, physical activity was found significantly associated with DFI. People who performed moderate-to-vigorous physical activity for 150–300 min per week or more were significantly less likely (AOR = 0.70, 95% CI: 0.53, 0.93; and AOR = 0.64, 95% CI: 0.52, 0.80) to experience DFI compared to people who performed similar physical activity for less than 150 min per week. Regarding health status variables, the study found a significant association of DFI with type-1 diabetes, longer diabetes duration, stroke, and asthma. Compared to people with type-1 diabetes, people with type-2 diabetes (AOR = 0.55, 95% CI: 0.33, 0.90) had lower odds of suffering from DFI. The likelihood of having DFI was significantly higher among people with diabetes duration 15 years or more (AOR = 1.62, 95% CI: 1.25, 2.10) compared to people with less than 5 years of diabetes duration. Results also indicated that people who had cardiovascular disease, had a stroke, or had asthma were more likely (AOR = 1.36, 95% CI: 1.11, 1.67, AOR = 1.4, 95% CI: 1.01, 1.94 and, AOR = 1.64, 95% CI: 1.26, 2.15, respectively) to have DFI compared to those who did not have the condition.

The revised AORs did not differ markedly from the complete case analysis (Supplementary Table 3), which indicated that missing data did not substantially affect the findings from the observed data.

## Discussion

This study found that 10.8% of adults with diabetes aged 45 years and older from NSW had DFD, 5.4% had DFU, while 5.2% had DFI during 2006–2012. The prevalence of DFD found in this study is higher than the recent pooled global prevalence of 7.1% [5]. However, the existing Australian studies suggested the DFD prevalence ranges from 7 to 15.1% [56, 57]. The prevalence of DFU found in our study is slightly higher than the recent pooled global prevalence of 4.6–4.8% [5, 6], although it is much lower compared to the prevalence found (15.1%) in an Australian inpatient study [57] in 2013. The variation in the prevalence can be attributed to the difference in population, geography, and/or time period.

The relationship between remoteness and diabetic foot complications is mixed in the literature [11, 19, 21, 35]. Our study demonstrated remoteness to be associated with DFD and DFU. These findings are similar to two of the few studies in Australia [11, 19] that had used the remoteness index. The Australian Institute of Health and Welfare (AIHW) study found that lower limb ulcer was prevalent among those who resided in rural areas [11]. However, the study did not distinguish whether the outcome was due to diabetes or not. Again, Singh (2018) concluded that the likelihood of attaining DFD was higher among people from remote areas using hospitalised patients’ data in Queensland, Australia [19]. On the other hand, our findings were in contradiction to some other Australian studies [21, 35] that found diabetic foot morbidity was less likely with an increase of rurality. Their studies were focused on regional areas where patients’ characteristics might have determined foot health rather than remoteness.

Previous studies had found ethnicity and country of birth as important factors for DFUs and DFIs [58, 59]. This study found that people born in Asia or in the Middle East had a lower likelihood of having DFD, DFU or DFI than people born in Australia, New Zealand, Canada, the UK, or the USA. Further, our study found that people born overseas had a lower likelihood of having diabetic foot complications, when a binary variable – whether born in Australia or not – was included in the analysis. Our result was comparable to a limited extent to the findings of the only Australian study that included the same binary variable in their analysis, and it was found that people born overseas had less likelihood of having peripheral neuropathy [20].

In this study, household income was significantly associated with DFD, DFU and DFI. The study found that people with higher income were less likely to suffer from any diabetic foot complications. Low income may limit access to additional health care services needed to manage foot health [17]. Similar to our findings, Nather et al. (2010) found that lower household income was associated with a higher likelihood of having diabetic foot among inpatients aged 24–91 years in Singapore. In another study based on emergency department setting in the USA during 2006–2010, Skrepnek et al. (2015) concluded that persons from the lowest income quartile regions had higher odds of major amputation compared to persons from highest-income regions [60]. The existing studies in Australia used socioeconomic indexes for areas (SEIFA) to measure socioeconomic status, which incorporated the average income of the population within a certain geography. In this regard, an Australian inpatient based study found that people residing in the relatively less disadvantaged area were significantly less likely to suffer from DFU [20]. However, our study suggested a result to the contrary of the Australian study, the reasons for which could probably be attributed to the fact that SEIFA might not represent an accurate measure of a person’s socioeconomic status [45]. Therefore, previous studies warranted further research to understand the association between individual-level socioeconomic status and diabetic foot complications [19]. Ours is the first in Australia to investigate the association of socioeconomic status with diabetic foot both at individual and aggregate level.

Another socioeconomic factor found to be associated with DFI was the possession of private health insurance. Our study found that patients with private health insurance had a lower likelihood of having DFI as opposed to those who did not have private health insurance. We, however, did not find any published Australian study comparable to our findings related to having private health insurance and diabetic foot complications. Our results, however, were in conformity to some other international studies, which found a significantly lower likelihood of diabetic amputation for private health insurance holders [38].

There were very few Australian studies that investigated the association of different lifestyle factors with diabetic foot complications. Two Australian based studies included smoking as a risk factor in their analysis [20, 22]. However, in one of these studies, Lazzarini et al. (2016) did not find any significant association between smoking and diabetic foot complications [20]. This was contrary to what we found in our study. However, our finding that an association between DFU and smoking exists was corroborated by other international studies [39, 61]. Although the mechanism of how it was related could not be comprehended, it was thought that smoking might promote low-density lipoprotein (LDL) oxidation to induce health issues [62]..

Further, our study found that DFD, DFU and DFI were reduced with the increase in moderate-to-vigorous physical activities. This finding corroborates Monica et al.’s (2018) systematic review findings that physical activity and exercise could improve diabetic foot related outcomes [63]. They reasoned that physical activity and exercise might significantly improve nerve velocity conduction, peripheral sensory function, and foot peak pressure distribution, resulting in a lower incidence of diabetic foot complications.

There exists robust evidence that the duration of diabetes is an important predictor of diabetes foot health [21, 22, 34], and it was a significant factor in our study even after controlling for age. It seems that existing Australian studies either did not assess the role of diabetes type on foot complications in the absence of clinical/ administrative data [34] or could not assess the association due to not having participants with all types of diabetes [22]. Many studies used diabetes type as a control variable but did not report the effect size [36, 64].

### Strengths and limitations

This study identified individuals with diabetes based on responses to self-reported surveys, emergency department presentation diagnosis codes and/or hospital admission. One of the strengths of this study is that the identification of diabetes is quite robust in this study compared to those studies which relied only on self-reported diabetes status or only used hospital admission diagnosis codes. Another strength of the study is its’ provision of insights into social factors related to DFD for the older population of NSW using a representative sample that was not previously explored.

The study has a few limitations as well. Firstly, the study could not ascertain the causal relationship between the study factors and the outcome variables due to the cross-sectional data collection. Secondly, the study could not rule out the possibility of underestimation of diabetes, and consequently DFDs in the absence of clinical data on diabetes during the survey. However, fact remains, clinical data is not always available for a large-scale study like the 45 and Up Study. Since the survey data were linked with administrative health data like APDC, EDDC and PBS, it is reasonable to infer that the information contained in the study could be of good use to ascertain a person’s diabetes status much better than those studies that were only based on self-reported data. Thirdly, the coding in ICD-10 AM, ICD-9 CM and SNOMED are prone to human error [65]. If the foot diseases were coded wrong or were not coded at all during admission, the estimate would result in underestimating DFD and its different types. However, one study reported that the accuracy of coding was acceptable to make reliable estimates [18]. Fourthly, the period to which our study related is not the most recent, but this does not diminish the strength of the findings. To our knowledge, this data is current for NSW because time-periods are associated with large data linkage-based studies due to the time involved in obtaining, training, linking, cleaning and validating cross-system data linkage. Lastly, there is a possibility of including leg ulcers in this study that were not precisely limited to the foot due to hospital coding. Despite these limitations, a major strength of the study is using a large representative survey linked with administrative health data that identifies individuals with diabetes and DFDs.

## Conclusion

This study demonstrated that, among others, remoteness and income were strong predictors of diabetic foot complications. Access to healthcare services may be inhibited by remote locations due to shortage of podiatrists and high costs of services. Low income may also adversely affect access to healthcare services and uptake of preventive measures as it incurs out-of-pocket costs. People with private health insurance can utilise more healthcare services as they may be able to have these costs reimbursed partially or fully. As a result, their likelihood of having diabetic foot complication decreases, as shown in our study. Therefore, provision of subsidised podiatry services for persons aged 45 and older with diabetes may be considered by the state or Federal health care services. Overall, policymakers should be aware that diabetic foot complications were unevenly distributed in the population. Therefore, the provision of health care services and policies should be designed in such a way so that inequalities do not hinder access to healthcare, thereby not contributing to increased complications among low-income groups or in disadvantaged areas. The study also suggests that high-risk patients, such as older adults, males, singles, individuals from English speaking countries, patients with type-1 diabetes and longer diabetes duration, should have a focused program promoting foot health.

## Supplementary Information

**Additional file 1.**

## Data Availability

Data contained in the 45 and Up Study cohort and other linked sources cannot be made publicly available due to privacy reasons. The policies and procedures of accessing data can be found at www.saxinstitute.org.au.

## Abbreviations

AIHW: Australian Institute of Health and Welfare
AOR: Adjusted Odds Ratio
APDC: Admitted Patient Data Collection
AUD: Australian Dollar
CHeReL: Centre for Health Record Linkage
CI: Confidence Interval
DFD: Diabetes-related foot disease
DFI: Diabetic foot infection
DFU: Diabetic foot ulcer
DVA: Department of Veterans’ Affairs
EDDC: Emergency Department Data Collection
ICD 9- CM: International Classification of Diseases 9 – Clinical Modification
ICD10-AM: International Classification of Diseases 10 – Australian Modification
NSW: New South Wales
PBS: Pharmaceutical Benefits Scheme
PVD: Peripheral vascular disease
SEIFA: Socioeconomic indexes for areas
SNOMED- CT: Systematized Nomenclature of Medicine - Clinical Terms
SURE: Secure Unified Research Environment
