# Supplementary material for: Social determinants of diabetes-related foot disease among older adults in New South Wales, Australia: evidence from a population-based study
Source: J Foot Ankle Res. 2021 Dec 16;14:65. doi: 10.1186/s13047-021-00501-8 (PMC8680161; doi:10.1186/s13047-021-00501-8)
Supplement: Supplementary file 1 — Additional file 1. [file 13047_2021_501_MOESM1_ESM.docx]

Supplementary Table 1: ICD-10 AM codes of diabetes-foot related complications

| **Complications** | **ICD-10 AM Codes** |
| --- | --- |
| Insulin-dependent diabetes mellitus  Non-insulin-dependent diabetes Mellitus  Other specified diabetes mellitus  Unspecified diabetes mellitus | E10  E11  E13  E14 |
| Diabetes mellitus with foot ulcer | E1x.73 |
| Ulcer of lower limb | L97 |
| Chronic skin ulcer of lower limb | L98.4 |
| Decubitus ulcer | L89 |
| Atherosclerosis of arteries of extremities with ulceration/gangrene | I70.23/I70.24 |
| Diabetes with peripheral angiopathy with gangrene | E1x.52 |
| Subsidiary of gangrene | R02x |
| Cellulitis | L03.02, L03.11 |
| Osteomyelitis | M86.x7, M86.x6 |
| Procedure codes (lower limb amputations, including foot, toe, and ankle) | 44370-00, 44373-00, 44367-01, 44367-02, 44338-00, 44358-00, 44361-00, 44361-01, 44364-00, 44364-01, 90557-00 |
| Diabetic neuropathic arthropathy (Charcot) | E1x.61 |
| Diabetic Mononeuropathy | G57, E1x.41 |
| Diabetic Peripheral neuropathy | E1x.42 |
| Diabetes with peripheral angiopathy without gangrene | E1x.51 |
| Other Peripheral circulatory complication | I73.9 |

Supplementary Table 2: ICD-9 CM codes of diabetes-foot related complications

| **Complications** | **ICD 9 CM codes** |
| --- | --- |
| Diabetes | 249, 250 |
| Ulcer of lower limb | 440.23, 707.1x |
| Gangrene of lower limb | 440.24, 785.4 |
| Arthropathy | 250.8, 713, 713.5 |
| Lower limb amputation | 84.1, 84.10–17 |
| Cellulitis | 680, 680.7, 681, 681.1, 681.10, 681.11, 681.9, 682, 682.6, 682.7 |
| Osteomyelitis | 730, 730.0, 730.1, 730.2, 730.9 |
| Peripheral Neuropathy | 250.6, 357.2 |
| Atherosclerosis | 440, 440.20–24, 440.29 |
| Peripheral vascular disease | 250.7, 443.8, 443.9, 997.2 |

Supplementary Table 3: Determinants of DFD, DFU and DFI (multiple imputation analysis)

|  | **Diabetes-related foot disease** | | | **Diabetic foot ulcer** | | | **Diabetic foot infection** | | |
| --- | --- | --- | --- | --- | --- | --- | --- | --- | --- |
| **Study factors** | **Adjusted OR** | **95% CI** | ***p* value** | **Adjusted OR** | **95% CI** | ***p* value** | **Adjusted OR** | **95% CI** | ***p* value** |
| **Demographic factors** |  |  |  |  |  |  |  |  |  |
| **Age** |  |  |  |  |  |  |  |  |  |
| 45-54 years | 1.00 |  |  | 1.00 |  |  | 1.00 |  |  |
| 55-64 years | 1.17 | (0.95 - 1.45) | 0.144 | 1.26 | (0.92 - 1.74) | 0.149 | 1.21 | (0.92 - 1.59) | 0.165 |
| 65-74 years | 1.52 | (1.23 - 1.88) | <0.001 | 1.55 | (1.14 - 2.13) | 0.006 | 1.24 | (0.93 - 1.64) | 0.139 |
| 75+ years | 2.65 | (2.14 - 3.29) | <0.001 | 3.18 | (2.33 - 4.34) | <0.001 | 1.99 | (1.49 - 2.65) | <0.001 |
| **Sex** |  |  |  |  |  |  |  |  |  |
| Male | 1.00 |  |  | 1.00 |  |  | 1.00 |  |  |
| Female | 0.66 | (0.59 - 0.74) | <0.001 | 0.67 | (0.57 - 0.78) | <0.001 | 0.62 | (0.53 - 0.73) | <0.001 |
| **Current Marital status** |  |  |  |  |  |  |  |  |  |
| Single | 1.00 |  |  | 1.00 |  |  | 1.00 |  |  |
| Married/defacto | 0.75 | (0.62 - 0.91) | 0.004 | 0.59 | (0.46 - 0.75) | <0.001 | 0.56 | (0.44 - 0.71) | <0.001 |
| Widowed | 0.98 | (0.79 - 1.22) | 0.881 | 0.81 | (0.61 - 1.07) | 0.131 | 0.83 | (0.64 - 1.09) | 0.184 |
| Divorced/separated | 1.01 | (0.80 - 1.28) | 0.907 | 0.89 | (0.66 - 1.20) | 0.447 | 0.74 | (0.55 - 1.00) | 0.048 |
| **Remoteness** |  |  |  |  |  |  |  |  |  |
| Major Cities | 1.00 |  |  | 1.00 |  |  | 1.00 |  |  |
| Inner Regional | 0.93 | (0.83 - 1.05) | 0.235 | 0.87 | (0.75 - 1.02) | 0.088 | 0.93 | (0.79 - 1.09) | 0.348 |
| Outer Regional | 0.93 | (0.81 - 1.08) | 0.365 | 0.76 | (0.62 - 0.93) | 0.009 | 1.05 | (0.86 - 1.28) | 0.636 |
| Remote | 1.55 | (1.10 - 2.17) | 0.011 | 1.22 | (0.77 - 1.94) | 0.394 | 1.65 | (1.05 - 2.58) | 0.029 |
| Very Remote | 0.46 | (0.16 - 1.33) | 0.151 | 0.91 | (0.31 - 2.68) | 0.863 | 0.70 | (0.20 - 2.40) | 0.568 |
| **Country of birth** |  |  |  |  |  |  |  |  |  |
| English Speaking countries | 1.00 |  |  | 1.00 |  |  | 1.00 |  |  |
| Europe | 0.84 | (0.73 - 0.97) | 0.015 | 0.77 | (0.63 - 0.94) | 0.009 | 0.82 | (0.67 - 1.01) | 0.064 |
| Middle East | 0.32 | (0.18 - 0.57) | <0.001 | 0.31 | (0.13 - 0.71) | 0.006 | 0.33 | (0.14 - 0.76) | 0.009 |
| Asia | 0.25 | (0.16 - 0.40) | <0.001 | 0.19 | (0.10 - 0.34) | <0.001 | 0.32 | (0.17 - 0.60) | <0.001 |
| Others | 0.53 | (0.36 - 0.79) | 0.002 | 0.37 | (0.21 - 0.66) | 0.001 | 0.45 | (0.24 - 0.83) | 0.010 |
| **Language spoken other than English** |  |  |  |  |  |  |  |  |  |
| No | 1.00 |  |  | 1.00 |  |  | 1.00 |  |  |
| Yes | 0.97 | (0.79 - 1.18) | 0.755 | 1.00 | (0.76 - 1.30) | 0.984 | 1.01 | (0.75 - 1.35) | 0.960 |

Supplementary Table 3: Determinants of diabetes-related foot disease, diabetic foot ulcer, and diabetic foot infection (multiple imputation analysis) (*Continued*)

|  | **Diabetes-related foot disease** | | | **Diabetic foot ulcer** | | | **Diabetic foot infection** | | |
| --- | --- | --- | --- | --- | --- | --- | --- | --- | --- |
| **Study factors** | **Adjusted OR** | **95% CI** | ***p* value** | **Adjusted OR** | **95% CI** | ***p* value** | **Adjusted OR** | **95% CI** | ***p* value** |
| **Socio-economic factors** |  |  |  |  |  |  |  |  |  |
| **Education** |  |  |  |  |  |  |  |  |  |
| Less than high school | 1.00 |  |  | 1.00 |  |  | 1.00 |  |  |
| High school certificate/ trade | 0.95 | (0.84 - 1.09) | 0.473 | 1.06 | (0.89 - 1.27) | 0.489 | 0.79 | (0.66 - 0.95) | 0.014 |
| Certificate/diploma | 0.97 | (0.84 - 1.12) | 0.654 | 0.91 | (0.73 - 1.12) | 0.365 | 1.01 | (0.82 - 1.23) | 0.952 |
| University degree or higher | 0.95 | (0.80 - 1.12) | 0.555 | 0.83 | (0.65 - 1.07) | 0.147 | 0.91 | (0.72 - 1.14) | 0.411 |
| **SEIFA (IRSD)** |  |  |  |  |  |  |  |  |  |
| quantile 1 (least disadvantaged) | 1.00 |  |  | 1.00 |  |  | 1.00 |  |  |
| quantile 2 | 1.12 | (0.96 - 1.31) | 0.144 | 1.15 | (0.92 - 1.42) | 0.218 | 1.18 | (0.95 - 1.45) | 0.130 |
| quantile 3 | 1.14 | (0.98 - 1.32) | 0.081 | 1.11 | (0.91 - 1.36) | 0.318 | 1.05 | (0.85 - 1.29) | 0.650 |
| quantile 4 | 1.16 | (1.00 - 1.36) | 0.056 | 1.05 | (0.85 - 1.30) | 0.674 | 1.07 | (0.86 - 1.33) | 0.528 |
| quantile 5 (Most disadvantaged) | 1.22 | (1.03 - 1.45) | 0.023 | 1.13 | (0.89 - 1.43) | 0.304 | 1.32 | (1.04 - 1.68) | 0.021 |
| **Annual Household income** |  |  |  |  |  |  |  |  |  |
| < AUD 20,000 | 1.00 |  |  | 1.00 |  |  | 1.00 |  |  |
| AUD 20,000 – <AUD 50,000 | 0.85 | (0.74 - 0.97) | 0.020 | 0.89 | (0.73 - 1.09) | 0.256 | 0.78 | (0.64 - 0.95) | 0.015 |
| >AUD 50,000 | 0.68 | (0.55 - 0.82) | <0.001 | 0.72 | (0.55 - 0.96) | 0.024 | 0.67 | (0.51 - 0.88) | 0.004 |
| **Private Health insurance** |  |  |  |  |  |  |  |  |  |
| No (without DVA/concession card) | 1.00 |  |  | 1.00 |  |  | 1.00 |  |  |
| No (with DVA/concession card) | 0.82 | (0.70 - 0.96) | 0.613 | 0.89 | (0.71 - 1.10) | 0.287 | 0.86 | (0.65- 1.10) | 0.685 |
| Yes | 0.99 | (0.86 - 1.14) | 0.872 | 1.06 | (0.87 - 1.29) | 0.559 | 0.75 | (0.51 - 0.84) | 0.030 |
| **Life-style factor** |  |  |  |  |  |  |  |  |  |
| **Ever being a regular smoker** |  |  |  |  |  |  |  |  |  |
| No | 1.00 |  |  | 1.00 |  |  | 1.00 |  |  |
| Yes | 1.30 | (1.17 - 1.44) | <0.001 | 1.26 | (1.09 - 1.45) | 0.001 | 1.08 | (0.93 - 1.25) | 0.303 |
| **Alcohol consumption** |  |  |  |  |  |  |  |  |  |
| <= 14 drinks per week | 1.00 |  |  | 1.00 |  |  | 1.00 |  |  |
| >14 drinks per week | 0.94 | (0.80 - 1.11) | 0.473 | 0.95 | (0.76 - 1.21) | 0.694 | 0.95 | (0.76 - 1.19) | 0.685 |

Supplementary Table 2: Determinants of diabetes-related foot disease, diabetic foot ulcer, and diabetic foot infection (multiple imputation analysis) (*Continued*)

|  | **Diabetes-related foot disease** | | | **Diabetic foot ulcer** | | | **Diabetic foot infection** | | |
| --- | --- | --- | --- | --- | --- | --- | --- | --- | --- |
| **Study factors** | **Adjusted OR** | **95% CI** | ***p* value** | **Adjusted OR** | **95% CI** | ***p* value** | **Adjusted OR** | **95% CI** | ***p* value** |
| **Total moderate-to-vigorous physical activity per week (minutes)** |  |  |  |  |  |  |  |  |  |
| <150 min | 1.00 |  |  | 1.00 |  |  | 1.00 |  |  |
| 150-300 min | 0.64 | (0.55 - 0.74) | <0.001 | 0.61 | (0.50 - 0.75) | <0.001 | 0.64 | (0.52 - 0.79) | <0.001 |
| >300 min | 0.59 | (0.53 - 0.66) | <0.001 | 0.52 | (0.45 - 0.60) | <0.001 | 0.64 | (0.55 - 0.74) | <0.001 |
| **Vegetables intake** |  |  |  |  |  |  |  |  |  |
| <5 serves per day | 1.00 |  |  | 1.00 |  |  | 1.00 |  |  |
| 5 or more serves per day | 1.02 | (0.92 - 1.14) | 0.682 | 1.03 | (0.89 - 1.20) | 0.655 | 1.10 | (0.94 - 1.28) | 0.218 |
| **Fruit intake** |  |  |  |  |  |  |  |  |  |
| <2 serves per day | 1.00 |  |  | 1.00 |  |  | 1.00 |  |  |
| 2 or more serves per day | 1.00 | (0.90 - 1.11) | 0.979 | 0.97 | (0.84 - 1.11) | 0.646 | 1.01 | (0.87 - 1.17) | 0.897 |
| **Health status factors** |  |  |  |  |  |  |  |  |  |
| **Type of diabetes** |  |  |  |  |  |  |  |  |  |
| Type-1 | 1.00 |  |  | 1.00 |  |  | 1.00 |  |  |
| Type-2 | 0.68 | (0.54 - 0.87) | 0.002 | 0.76 | (0.54 - 1.06) | 0.103 | 0.64 | (0.45 - 0.91) | 0.012 |
| **Duration of diabetes** |  |  |  |  |  |  |  |  |  |
| <5 years | 1.00 |  |  | 1.00 |  |  | 1.00 |  |  |
| 5 to <10 years | 1.30 | (1.09 - 1.55) | 0.004 | 1.28 | (0.98 - 1.66) | 0.068 | 1.12 | (0.89 - 1.40) | 0.341 |
| 10 to <15 years | 1.53 | (1.27 - 1.83) | <0.001 | 1.56 | (1.22 - 2.00) | <0.001 | 1.23 | (0.97 - 1.57) | 0.090 |
| 15 years or more | 2.20 | (1.86 - 2.62) | <0.001 | 2.51 | (1.97 - 3.20) | <0.001 | 1.77 | (1.42 - 2.22) | <0.001 |
| **BMI classification** |  |  |  |  |  |  |  |  |  |
| <18.5 | 1.00 |  |  | 1.00 |  |  | 1.00 |  |  |
| 18.5 to less than 25 | 0.55 | (0.37 - 0.84) | 0.005 | 0.52 | (0.32 - 0.85) | 0.009 | 0.64 | (0.36 - 1.13) | 0.124 |
| 25 to less than 30 | 0.55 | (0.37 - 0.83) | 0.005 | 0.39 | (0.24 - 0.64) | <0.001 | 0.67 | (0.38 - 1.18) | 0.166 |
| 30 or more | 0.65 | (0.43 - 0.98) | 0.038 | 0.53 | (0.33 - 0.86) | 0.010 | 0.97 | (0.55 - 1.70) | 0.911 |
| **High Blood Pressure** |  |  |  |  |  |  |  |  |  |
| No | 1.00 |  |  | 1.00 |  |  | 1.00 |  |  |
| Yes | 0.92 | (0.83 - 1.02) | 0.113 | 0.86 | (0.74 - 0.99) | 0.034 | 0.87 | (0.76 - 1.01) | 0.063 |

Supplementary Table 2: Determinants of diabetes-related foot disease, diabetic foot ulcer, and diabetic foot infection (multiple imputation analysis) (*Continued*)

|  | **Diabetes-related foot disease** | | | **Diabetic foot ulcer** | | | **Diabetic foot infection** | | |
| --- | --- | --- | --- | --- | --- | --- | --- | --- | --- |
| **Study factors** | **Adjusted OR** | **95% CI** | ***p* value** | **Adjusted OR** | **95% CI** | ***p* value** | **Adjusted OR** | **95% CI** | ***p* value** |
| **High Blood Cholesterol** |  |  |  |  |  |  |  |  |  |
| No | 1.00 |  |  | 1.00 |  |  | 1.00 |  |  |
| Yes | 0.87 | (0.78 - 0.97) | 0.014 | 0.88 | (0.75 - 1.03) | 0.106 | 0.86 | (0.73 - 1.00) | 0.055 |
| **Cardiovascular disease** |  |  |  |  |  |  |  |  |  |
| No | 1.00 |  |  | 1.00 |  |  | 1.00 |  |  |
| Yes | 1.49 | (1.34 - 1.66) | <0.001 | 1.37 | (1.18 - 1.58) | <0.001 | 1.36 | (1.17 - 1.57) | <0.001 |
| **Stroke** |  |  |  |  |  |  |  |  |  |
| No | 1.00 |  |  | 1.00 |  |  | 1.00 |  |  |
| Yes | 1.08 | (0.92 - 1.26) | 0.332 | 1.04 | (0.84 - 1.28) | 0.737 | 1.09 | (0.88 - 1.35) | 0.425 |
| **Asthma** |  |  |  |  |  |  |  |  |  |
| No | 1.00 |  |  | 1.00 |  |  | 1.00 |  |  |
| Yes | 1.12 | (0.97 - 1.29) | 0.130 | 0.96 | (0.79 - 1.17) | 0.676 | 1.39 | (1.16 - 1.66) | <0.001 |
| **Psychological distress** |  |  |  |  |  |  |  |  |  |
| None/low/moderate | 1.00 |  |  | 1.00 |  |  | 1.00 |  |  |
| High/very high | 1.36 | (1.16 - 1.59) | <0.001 | 1.20 | (0.96 - 1.50) | 0.108 | 1.22 | (0.98 - 1.52) | 0.075 |

Note: OR= Unadjusted Odds Ratio; AOR= Adjusted Odds ratio; SEIFA: Socio-Economic Indexes for Areas; IRSD: The Index of Relative Socio-Economic Disadvantage; DVA: Department of Veterans’ Affairs; Psychological distress, None/low/moderate: less than 22 Kessler-10 (K10) score
